# Prioritizing biomaterials for spinal disc implants by a fuzzy AHP and TOPSIS decision making method

**DOI:** 10.1038/s41598-023-48735-9

**Published:** 2023-12-06

**Authors:** Hossein Ansaripour, Kim Lars Haeussler, Stephen J. Ferguson, Markus Flohr

**Affiliations:** 1 CeramTec GmbH, CeramTec-Platz 1-9, 73207 Plochingen, Germany; 2 Institute for Biomechanics, D-HEST, ETH Zurich, Gloriastrasse 37 / 39, 8092 Zurich, Switzerland

**Keywords:** Biomedical engineering, Mechanical engineering, Biomaterials, Mathematics and computing

## Abstract

Considerable research has been focused on identifying the optimum biomaterial for spine implants. New technologies and materials have allowed surgeons to better grasp the biomechanical principles underpinning implant stability and function. An optimal biomaterial for total disc replacement (TDR) should include essential characteristics such as biocompatibility, long-term durability, the capacity to withstand mechanical stresses, and economic viability. Our research has focused on six biomaterials for TDR, including Ti–6Al–4V, CoCr alloy, stainless steel 316L, zirconia toughened alumina (ZTA), polyether ether ketone (PEEK) and ultra-high-molecular weight polyethylene (UHMWPE). Ten common properties, i.e., the Young’s modulus, density, tensile strength, the expense of the manufacturing process, the cost of raw material, wear rate, corrosion resistance, thermal conductivity, fracture toughness and compressive strength were utilized to assess these six different materials. The purpose of this study was to evaluate and rank the six alternative biomaterials proposed for use in the endplates and articulating surface of a spinal TDR. To accomplish this, a multi-criteria decision-making approach, namely the fuzzy analytic hierarchy process (fuzzy AHP) and the Technique of Order Preference by Similarity to Ideal Solution (TOPSIS) was adopted to solve the model. For validation and robustness of the proposed method, sensitivity analysis was performed, and comparison was performed with fuzzy-VIKOR and fuzzy-MOORA methods. In light of the study’s results, ZTA and Ti–6Al–4V were identified as the best suited materials for the articulating surface and endplates, respectively, in a spinal disc implant.

## Introduction

The first lumbar disc replacement in 1960 was a steel ball inserted between two vertebrae, which resulted in several postoperative complications^1^. In the 1980s, implants evolved from a stainless steel ball to two steel or titanium plates with a polyethylene sliding core in between^2^. The SB Charite prothesis was an evolution of such an implant, consisting of two chromium–cobalt plates and a mobile polyethylene core, with the aim to mimic the natural kinematics of the disc^3^. Plates with a central titanium stem were incorporated into the ProDisc-L, developed in 1989^4^. For the cervical site, the ProDisc-C implant was developed, consisting of a UHMWPE core and CoCr alloy endplates combined with a rough titanium surface coating to promote bone growth^5^. The Mobi-C cervical disc prosthesis was a further innovation, consisting of three components: two metal plates (composed of CoCr alloy) covered with a hydroxyapatite coating (to facilitate bone grafting) and a polyethylene plate in the center^6^. More recently, TDRs with ceramic components have been introduced due to its great wear resistance and biocompatibility^7^.

Current total disc replacements (TDR) seek to restore mobility and quality of life^8^. From a meta-analysis, TDRs had a relatively low rate of complications after 5-years for lumbar TDR (0–16.7%) and cervical TDR (0–4.0%)^9^. Nonetheless, Virk et al.^10^ conducted a cross-sectional analysis of TDR complications by querying the MAUDE (Manufacture and User Facility Device Experience) database and alternative summary reporting (ASR) data. Migration, insertion issues, neck pain, heterotopic ossification, and radiculopathy were identified as complications. Some of these issues may be attributable to the materials utilized to construct TDRs.

TDRs require materials to demonstrate biocompatibility and biostability, but the challenge of finding an optimal material lies in identifying a material with the appropriate Young’s modulus, stiffness, wear rate, corrosion, and fatigue resistance, to name just a few characteristics^11^. Accurate material selection based on established criteria is challenging, and we are frequently confronted with a plethora of options while making a decision. For instance, osseointegration is one of the desired properties of biomaterials, as it enables the implant to bond with the surrounding host bone. However, if this material is used for articulating surfaces, it can stimulate heterotopic ossification and impair its function. Furthermore, the chosen materials must lead to technological solutions that are affordable and can be put into practice with reasonable expenditure on materials development and manufacturing of products required by the application.

Multi-criteria decision-making (MCDM) provides a strategy for addressing difficulties involving the selection from a finite number of alternatives, including those with the same attributes^12^. This method determines how attribute data should be handled to reach a solution^13^. MCDM is divided into three parts: selection of alternatives and criteria, determination of weight criteria, and ranking of alternatives^14^. Several popular options including weighted product method (WPM), technique for order preference by similarity to ideal solution (TOPSIS), Vise Kriterijumska Optimizacija Kompromisno Resenje (VIKOR) method, analytical hierarchy process (AHP), complex proportional assessment (COPRAS) and multi-objective optimization on the basis of ratio analysis (MOORA), and the preference ranking organization method for enrichment evaluations (PROMETHEE), were used in previous studies^15–24^. Recently, multi-criteria decision analysis has been widely utilized in numerous scientific fields, including product design, transportation, manufacturing, human resource management, quality control, marine application, and renewable energy^25–27^. Sen et al.^28^ used Type-2 fuzzy AHP-ARAS (additive ratio assessment) to select the best parametric combination of the wire electrical discharge machining. The objective of their research was to minimize costs and human effort associated with the machining process of nickel-based alloys^28^. Hussain et al.^29^ developed a robust MCDM considering the non-deterministic nature of decision maker along with the vagueness in decision. They assessed ratings of alternatives versus criteria using parametric interval valued intuitionistic fuzzy number (PIVIFN)^29^. In their proposed model, the aggregated decision matrix was converted into a matrix which indicated the relative benefit for not selecting the alternative with the lowest benefit or highest cost^29^. The other study employed AHP in conjunction with COPRAS and TOPSIS to determine the optimal type of carbon nanotube under grey environment^30^. They stated that different methodologies may show different outcomes. Therefore, validation of the results is required for making the decision^30^. Yadav et al.^31^ proposed the hybrid preference selection index (PSI)-TOPSIS approach for effective material selection in marine applications. According to their findings, the PSI method is relevant when there is difficulty in assessing the relative importance of variables and the TOPSIS method proficiently deals with the physical attributes and the number of available alternatives^31^. Gangwar et al.^32^ used an adaptive neuro-fuzzy inference system (i.e., a combination of fuzzy logic and neural networks) to find the optimal combination of reinforcement materials enhancing wear resistance.

Chowdary et al.^21^ also used the combination of fuzzy AHP-TOPSIS to assess and rank some alternative materials for biomedical engineering applications, including joint replacement, bone plates, and dental implants. Yadaw et al.^33^ performed hybrid AHP-TOPSIS to predict the best formulation of dental restorative composite materials. The other study used PSI as an MCDM method to identify the optimal formulation and ranking of ceramic particulates for dental restorative composite materials^34^. The authors also suggested that the utilization of PSI can be advantageous for material scientists when making decisions, as it helps address the inherent conflicts arising from diverse material sets^34^. The FAHP-FTOPSIS, Entropy-VIKOR, and AHP-MOORA methods were also employed as novel approaches to ascertain the weight criteria and rank the alternatives of dental restorative composite materials^35–37^.

In the review of available and related literature, very few studies on material selection in biomedical engineering applications were found. Moreover, biomedical applications serve separate and distinct functions. For better prioritization, it is suggested that biomaterials be classified uniquely for each application. For instance, TDR requires both good articulation and anchorage to vertebrae. Moreover, the selection process extends beyond merely considering quantitative criteria. By incorporating qualitative parameters alongside quantitative ones, a more comprehensive evaluation can be achieved.

This study aimed to implement a decision-making process for the biomaterial selection of endplates and articulating surfaces in spinal disc prosthesis. In this context, a combination of analytical hierarchy process (AHP) and fuzzy set was used to specify the relative importance of the evaluation criteria. The precise specification of criteria importance was fine-tuned to the unique requirements of endplates and articulating surfaces. In line with the complex and nuanced demands of spinal disc prosthesis, we also integrated qualitative criteria into our approach, enabling a more thorough assessment. The TOPSIS approach then ranked the candidate materials based on their weighted criteria derived from the fuzzy AHP process. Furthermore, sensitivity analysis was performed, and a comparison was conducted with fuzzy-VIKOR and fuzzy-MOORA methods to substantiate the reliability and consistency of this decision-making process's outcomes.

## Materials and methods

### Fuzzy analytical hierarchy process (fuzzy AHP)

The AHP process is a decision-making technique designed to solve problems by decomposing them, grouping them, and then organizing them hierarchically. The method involves a comparison of criteria paired with a predetermined measuring scale to identify priority criteria. Since the primary input of the AHP approach is the perception of the experts, retrieval decisions are subjective. This technique additionally considers consistency of data with inconsistency bounds^38^. However, the accuracy of data and, consequently, the results will be impacted by a high degree of uncertainty and doubt in the evaluation process. Hence, the AHP process was extended based on the fuzzy logic theory. The fuzzy AHP approach is utilized similarly to the AHP method. The only difference is that the fuzzy AHP method transforms the AHP scale into a fuzzy triangle scale for priority access. The following stages were taken sequentially for the development of the fuzzy AHP method.

#### Define the problem

**Figure 1:**
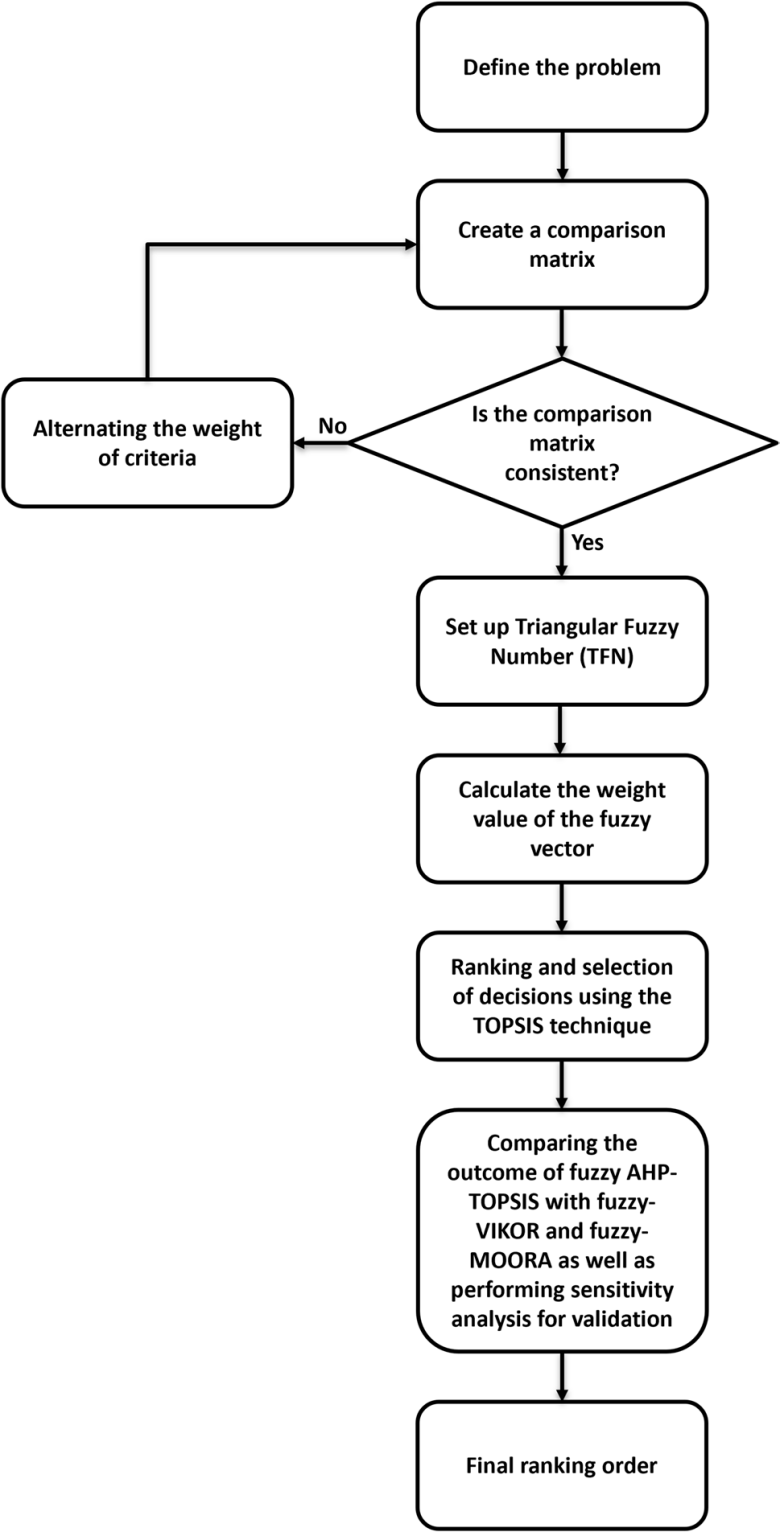
Decision-making process.

The problem was defined according to the criteria used to determine an appropriate material for endplates and articulating surfaces in TDR. In general, Fig. 1 illustrates the workflow of the decision-making process utilized in this paper. In this study, six biomaterials were considered as alternatives for TDR, including Ti–6Al–4V, CoCr alloy, stainless steel 316L, zirconia toughened alumina (ZTA), polyether ether ketone (PEEK), and ultra-high-molecular weight polyethylene (UHMWPE). Ten common properties, including the Young’s modulus (GPa), density (g/cm^3^), tensile strength (MPa), the expense of the manufacturing process (qualitative), the cost of raw material (qualitative), wear rate (qualitative), corrosion resistance (qualitative), thermal conductivity (W/mK), fracture toughness (Mpa√m), and compressive strength (MPa), were considered the main criteria for material selection in TDR applications (Table 1). The quantitative data was extracted from published sources^21, 39–46^. Due to a lack of comprehensive data and variations in measuring and testing methodologies for wear and corrosion studies, qualitative data was assigned on a numeric scale to these parameters^47–53^. According to Table 1, the lowest and highest values demonstrate the best wear rate and corrosion resistance performance for each material, respectively. Typically, manufacturers ascertain the costs associated with the manufacturing process. In addition to additive manufacturing, casting, extrusion, CNC, and injection molding are among the many procedures available. For instance, certain manufacturers might select the expensive injection molding method for production, whereas others might opt for the comparatively less expensive additive manufacturing method. The cost of raw materials is also established by the suppliers, and the quotations provided by different suppliers may differ, thereby complicating the task of determining an exact cost for each material. As a consequence, we consulted with some specialists at CeramTec and endeavored to assign a realistic metric value to these criteria. The highest value in Table 1 corresponds to the highest cost. It is worth noting that the material properties in Table 1 particularly for ZTA were based on published data and there was no evidence that this ZTA was used for implants. However, these information are useful for design guidance.

**Table 1:** Objective data of the attributes of alternative biomaterials for TDR application^21, 39–45^.

Material	E (GPa)	Ρ (g/cm^3^)	Tensile strength (MPa)	Expense of manufacturing process (qualitative)	Cost of raw material (qualitative)	Wear rate (qualitative)	Corrosion resistance (qualitative)	Thermal conductivity (w/mK)	Fracture toughness (MPa/√m)	Compressive strength (MPa)
ZTA	338	4.30	350	5	2	1	5	24	6	2758
CoCr alloy	220	8.77	1403	3	4	2	3	14.8	100	1296
Ti–6Al–4V	114	4.42	940	3	3	3	4	7.2	91	1172
Stainless steel 316L	193	8	485	2	1	4	2	16.3	95	620
UHMWPE	1	0.95	21.4	2	1	5	5	0.48	6.41	13.8
PEEK	3.6	1.32	80	3	5	5	5	0.25	6.76	124

E and ρ represent the Young’s modulus and density, respectively. For the qualitative parameters^47–53^, the highest intensity is 5, while the lowest intensity is 1.

#### Create a comparison matrix

The pair-wise comparison matrix is simple, has a strong position for the consistency framework, obtains other information that may be required with all possible comparisons, and is able to analyze the overall priority sensitivity for changes in consideration. Equation (1) is utilized to define pair-wise comparisons.

1 $${a}_{ij}= \frac{{w}_{i}}{{w}_{j}}, i,j=1, 2,\dots , n,$$

where w_i_ and w_j_ are weights for the criterion i and the criterion j, n denotes the number of compared criteria, and a_ij_ is the ratio of criterion i’s weight to criterion j’s weight. The scale of relative relevance for constructing a pair-wise comparison matrix is depicted in Table 2. Table 3 indicates the pair-wise comparison matrix constructed in this paper. The following rules were taken into account when constructing the pair-wise comparison matrix:

**Table 2:** Scale of relative importance in AHP scale and fuzzy AHP scale.

Definition	Intensity of relative importance in AHP scale	Intensity of relative importance in triangular fuzzy number (TFN) scale
Equal importance	1	(1,1,1)
Moderate importance	3	(2,3,4)
Strong importance	5	(4,5,6)
Very strong importance	7	(6,7,8)
Extreme strong importance	9	(9,9,9)
Intermediate values	2	(1,2,3)
	4	(3,4,5)
	6	(5,6,7)
	8	(7,8,9)

**Table 3:** The AHP pair-wise comparison matrix for design of (a) the articulating surface and (b) the endplate.

	C1	C2	C3	C4	C5	C6	C7	C8	C9	C10
(a)										
C1	1.00	5.00	3.00	2.00	2.00	0.20	0.20	7.00	2.00	1.00
C2	0.20	1.00	0.20	0.16	0.16	0.13	0.13	4.00	0.20	0.18
C3	0.33	5.00	1.00	2.00	2.00	0.18	0.18	7.00	0.60	0.50
C4	0.50	6.25	0.50	1.00	1.00	0.20	0.20	7.00	0.50	0.50
C5	0.50	6.25	0.50	1.00	1.00	0.20	0.20	7.00	0.50	0.50
C6	5.00	7.69	5.55	5.00	5.00	1.00	1.00	8.00	5.00	5.00
C7	5.00	7.69	5.55	5.00	5.00	1.00	1.00	8.00	5.00	5.00
C8	0.14	0.25	0.14	0.14	0.14	0.13	0.13	1.00	0.14	0.14
C9	0.50	5.00	1.67	2.00	2.00	0.20	0.20	7.14	1.00	0.50
C10	1.00	5.55	2.00	2.00	2.00	0.20	0.20	7.14	2.00	1.00
(b)										
C1	1.00	7.00	4.00	5.00	5.00	8.00	1.00	7.00	4.00	4.00
C2	0.14	1.00	0.20	0.16	0.16	5.00	0.13	4.00	0.20	0.18
C3	0.25	5.00	1.00	2.00	2.00	7.00	0.18	7.00	0.60	0.50
C4	0.20	6.25	0.50	1.00	1.00	5.00	0.20	7.00	0.50	0.50
C5	0.20	6.25	0.50	1.00	1.00	5.00	0.20	7.00	0.50	0.50
C6	0.13	0.20	0.14	0.20	0.20	1.00	0.13	0.50	0.18	0.18
C7	1.00	7.69	5.55	5.00	5.00	7.69	1.00	8.00	5.00	5.00
C8	0.14	0.25	0.14	0.14	0.14	2.00	0.125	1.00	0.14	0.14
C9	0.25	5.00	1.67	2.00	2.00	5.55	0.20	7.14	1.00	0.50
C10	0.25	5.55	2.00	2.00	2.00	5.55	0.20	7.14	2.00	1.00

In these tables, C1 = Young’s modulus, C2 = density, C3 = tensile strength, C4 = expense of manufacturing process, C5 = cost of raw material, C6 = wear rate, C7 = corrosion resistance, C8 = thermal conductivity, C9 = fracture toughness, and C10 = compressive strength.

- If the element on the left of the pair-wise comparison matrix is more important than the element on the right, a positive integer (from 1 to 9) is placed in the cell; conversely, the reciprocal value of the integer is entered (Table 3).
- The relative importance of each element relative to itself is one; hence, the diagonal of the matrix contains only ones (Table 3).

After determining the comparison of its criterion, each column is normalized into matrix form by dividing each value in column i and row j by the sum of its column (see Eq. 2).

2 $${a}_{ij}= \frac{{a}_{ij}}{\sum {a}_{ij}} \forall i,j.$$

#### Consistency evaluation

To calculate the value of consistency, the eigenvector, which is the weighted value of the criterion, must be first recognized. The eigenvector is calculated by Eq. (3):

3 $${w}_{eigvec}=\frac{{\hat{a} }_{i}}{n} , \forall i,$$

where w_eigvec_ is the eigenvector, â_i_ is the sum of the matrix normalization values in each row and is divided by the number of criterion (n). Then the largest eigenvalue (λ_max_) is obtained by multiplying the number of columns with the main eigenvector (w_eigvec_) (Eq. 4). Then the consistency index and consistency ratio are calculated by Eqs. (5) and (6).

4 $${\lambda }_{max}=\sum_{j}(\sum_{i}{a}_{ij}\times {{w}_{eigvec}}_{j}) , \forall i,j,$$

5 $$CI = \frac{{\lambda }_{max}-n}{n-1},$$

6 $$CR =\frac{CI}{RI},$$

where CI and CR denote the consistency index and consistency ratio, respectively. To obtain the consistency ratio (CR), CI is divided by the ratio index RI (see Table 4) for the same sized matrix. Saaty et al.^38^ provided a comparison of the consistency index with a ratio index (RI) value (Table 4). This value relies on the order of the matrix (n). It is worth mentioning that CR should be around 10% or less to be acceptable. If the CR falls outside of this range, the participants’ evaluations should be revised (Fig. 1).

**Table 4:** Ratio index (RI)^38^.

n	1	2	3	4	5	6	7	8	9	10
RI	0	0	0.58	0.9	1.12	1.24	1.32	1.41	1.45	1.49

#### Triangular fuzzy number (TFN)

In general, two primary types of fuzzy membership functions are commonly employed, namely triangular and trapezoidal. Among them, triangle membership functions are frequently favored. The main rationale for utilizing triangular fuzzy sets over trapezoidal ones is in their inherent simplicity and ease of computation^27, 54^. Moreover, while dealing with subjective and imprecise information, the utilization of the triangular fuzzy set demonstrates its efficacy in developing decision-making problems^27, 54^.

**Figure 2:**
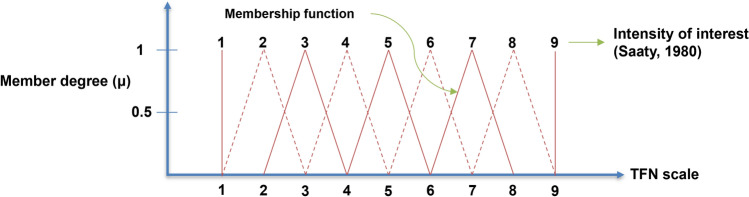
Triangular fuzzy set.

The fuzzy AHP scale has three values, namely, the lowest value (lower, L), the middle value (median, M), and the highest value (upper, U) (Fig. 2). The AHP comparison value is transformed to the fuzzy AHP scale value considering the scale of relative importance in Table 2 and according to the following equations:

7 $$a_{ij} \;in\;Fuzzy\; AHP = \left\{ {\begin{array}{*{20}l} {\left( {a_{ij} - 1,a_{ij} ,a_{ij} + 1} \right)} \hfill & {a_{ij} \;in\; AHP \; > \;1} \hfill \\ {\left( {\frac{1}{{integer\left( {\frac{1}{{a_{ij} }}} \right) + 1}},\frac{1}{{integer\left( {\frac{1}{{a_{ij} }}} \right)}},\frac{1}{{integer\left( {\frac{1}{{a_{ij} }}} \right) - 1}}} \right)} \hfill & {0\; < \;a_{ij} \;in\; AHP\; < \;1} \hfill \\ \end{array} } \right..$$

#### Calculate the weight value of the fuzzy vector

According to the aggregated pair-wise comparison matrix, the geometric mean (r) for the i_th_ criterion is calculated as follows:

8 $${r}_{i}=\bigg({\prod_{j=1}^{m}{a}_{ij}\bigg)}^\frac{1}{m}.$$

${r}_{i}$ represents the fuzzy geometric mean value for the i_th_ criterion, which is calculated by multiplying the fuzzy numbers (${a}_{ij}$ according to Eq. 7) in each row (i.e., lower values multiplied by lower values, middle values multiplied by middle values, and higher values multiplied by higher values in each row). The mean value is then rooted by the number of criteria in each row (m). Then the fuzzy weight (W_F_) for the i_th_ criterion is calculated as follows:

9 $${W}_{Fi}= {r}_{i} \times {(\sum_{i=1}^{n}{r}_{i})}^{-1}.$$

To calculate the weight factor of each criterion (${W}_{Fi}$), first all geometric mean values (r_i_) are summed together (i.e., lower values summed together, middle values summed together, and upper values summed together). The resultant value is then reciprocated and multiplied by the fuzzy geometric mean value of each criterion (${r}_{i}$).To get a crisp numerical value for fuzzy weights (${W}_{Fi}$), the de-fuzzification approach is conducted by calculating the center of area (COA):

10 $${W}_{Fi}=\left({L}_{i},{M}_{i},{U}_{i}\right),$$

11 $$DEFuzz{y}_{weight}=COA= \frac{{L}_{i}+{M}_{i}+{U}_{i}}{3}.$$

Finally, the normalized weight is computed through dividing the weight factor of each criterion by the sum of the weight factors.

### TOPSIS

The logic of TOPSIS is based on the concept that the selected option should have the shortest geometric distance from the best solution and the furthest geometric distance from the worst solution^55^. The TOPSIS process can be encapsulated in six steps as follows.

*Step 1* The evaluation matrix (A) is created according to the M alternatives (biomaterials in Table 1) and N attributes (parameters in Table 1):

12 $${A=({a}_{ij})}_{M\times N}.$$

*Step 2* The evaluation matrix is normalized by dividing the value of each criterion (${a}_{ij}$) by the root sum squared of the criteria in each column as follows:

13 $${\alpha }_{ij}=\frac{{a}_{ij}}{\sqrt{\sum_{i=1}^{M}{{(a}_{ij})}^{2}}}.$$

*Step 3* The weighted normalized decision matrix is obtained by multiplying the normalized decision matrix (${\alpha }_{ij}$) by its associated normalized fuzzy AHP weights (${w}_{j}$):

14 $${X}_{ij}={\alpha }_{ij}\times {w}_{j}.$$

*Step 4* Determine the best (b) and worst (w) alternative (A) for each attribute ($X$):

15a $${X}_{j}^{b}={Max}_{i=1}^{A}{X}_{ij},\, if\, the\, maximum\, value\, is\, a\, desired \,value,$$

15b $${X}_{j}^{b}={Min}_{i=1}^{A}{X}_{ij},\, if\, the\, minmum \,value \,is\, a \,desired\, value,$$

16a $${X}_{j}^{w}={Min}_{i=1}^{A}{X}_{ij}\, if\, the\, minimum\, value\, is \,not\, a \,desired \,value,$$

16b $${X}_{j}^{w}={Max}_{i=1}^{A}{X}_{ij}\, if\, the\, maximum\, value\, is\, not\, a\, desired\, value.$$

*Step 5* The Euclidean distance between the target alternative (${X}_{ij}$) and the best (${X}_{j}^{b}$) and worst (${X}_{j}^{w}$) alternative is respectively calculated:

17 $${D}_{i}^{b}=\sqrt{\sum_{j=1}^{N}{({X}_{ij}-{X}_{j}^{b})}^{2}},$$

18 $${D}_{i}^{w}=\sqrt{\sum_{j=1}^{N}{({X}_{ij}-{X}_{j}^{w})}^{2}.}$$

${D}_{i}^{b}$ denotes the Euclidean distance between the target alternative and the best alternative, and ${D}_{i}^{w}$ indicates the Euclidean distance between the target alternative and the worst alternative.

*Step 6* The relative closeness (S_i_) to the ideal solution is calculated and the performance order is ranked. The relative closeness of each alternative can be expressed as:

19 $${S}_{i}= \frac{{D}_{i}^{w}}{{D}_{i}^{b}+{D}_{i}^{w}}.$$

S_i_ lies between 0 and 1, and the greatest value means the better performance of the alternatives. Hence, the highest relative closeness value has been taken as the best alternative for endplate and articulating surface applications.

### Sensitivity analysis and method validation

A sensitivity analysis of the results was conducted by varying the weights of each criterion in order to determine its impact on ranking. It also reveals whether the ranking orders remain consistent despite weight criteria variations. In addition to fuzzy AHP, four more possible ways were considered to identify the weight factors of criteria. Initially, equal weight factors were considered for all criteria. Afterwards, the criteria were divided into three groups (i.e., most beneficial, beneficial, and least beneficial criteria). These classifications were made as each material property has a unique effect on the performance of an articulating surface or endplate. Table 5 shows how both articulating surface and endplate case studies are grouped. Sequentially, the weight factors were assigned as follows:

- 33% most beneficial, 33% beneficial, and 33% least beneficial criteria.
- 40% most beneficial, 40% beneficial, and 20% least beneficial criteria.
- 50% most beneficial, 40% beneficial, and 10% least beneficial criteria.

**Table 5:** Classification of criteria according to their level of importance for the function of the articulating surface and endplate.

	Most beneficial criteria	Beneficial criteria	Least beneficial criteria
Articulating surface	Wear rate Corrosion resistance	Young’s modulus Tensile strength Expense of manufacturing process Cost of raw material Fracture toughness Compressive strength	Density Thermal conductivity
Endplate	Young’s modulus Corrosion resistance	Tensile strength Expense of manufacturing process Cost of raw material Fracture toughness Compressive strength	Density Wear rate Thermal conductivity

To validate the potential of hybrid fuzzy AHP-TOPSIS methodology in suitable biomaterial selection, the results were also compared with fuzzy-VIKOR^56^ and fuzzy-MOORA^57^ methodologies.

### Postprocessing

Multi-criteria decision analysis (i.e., fuzzy AHP-TOPSIS) was used to determine the material for the design of endplate and bearing surfaces in TDR. This is a useful tool that may be applied to a variety of intricate decisions. Case studies determine the structure of the comparison matrix in the fuzzy AHP process. Wear rate and corrosion resistance are most relevant for articulating surfaces, while Young’s modulus and corrosion resistance are most important for the endplates (Table 3). Since density and thermal conductivity were assessed to be of moderate importance to the designs, they were assigned the lowest relative weights (Table 3). The other attributes, which play a major role in TDR design, were assigned strong relative importance (Table 3).

In the TOPSIS process, the attributes including Young’s modulus, density, expense of manufacturing process, the cost of raw material, and wear rate are considered beneficial with lower values, while the others, including tensile strength, corrosion resistance, thermal conductivity, fracture toughness, and compressive strength, are advantageous with higher values (Table 1). These specifications enable the determination of the best and worst alternatives for each attribute.

**Figure 3:**
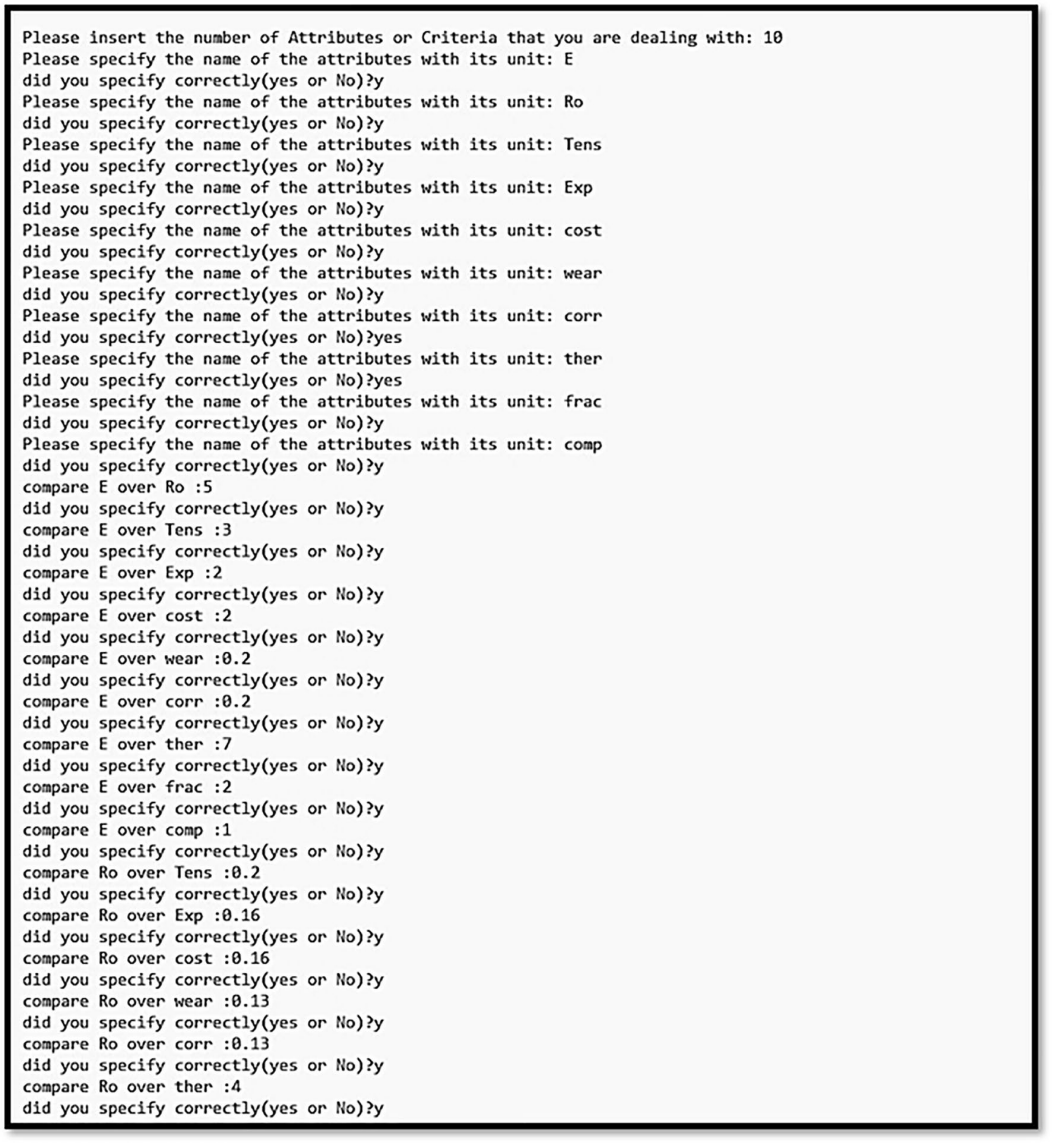
The initial segment of the Python function requests the number of attributes and the relative importance of each attribute in relation to the others so that a pair-wise comparison matrix can be generated.

**Figure 4:**
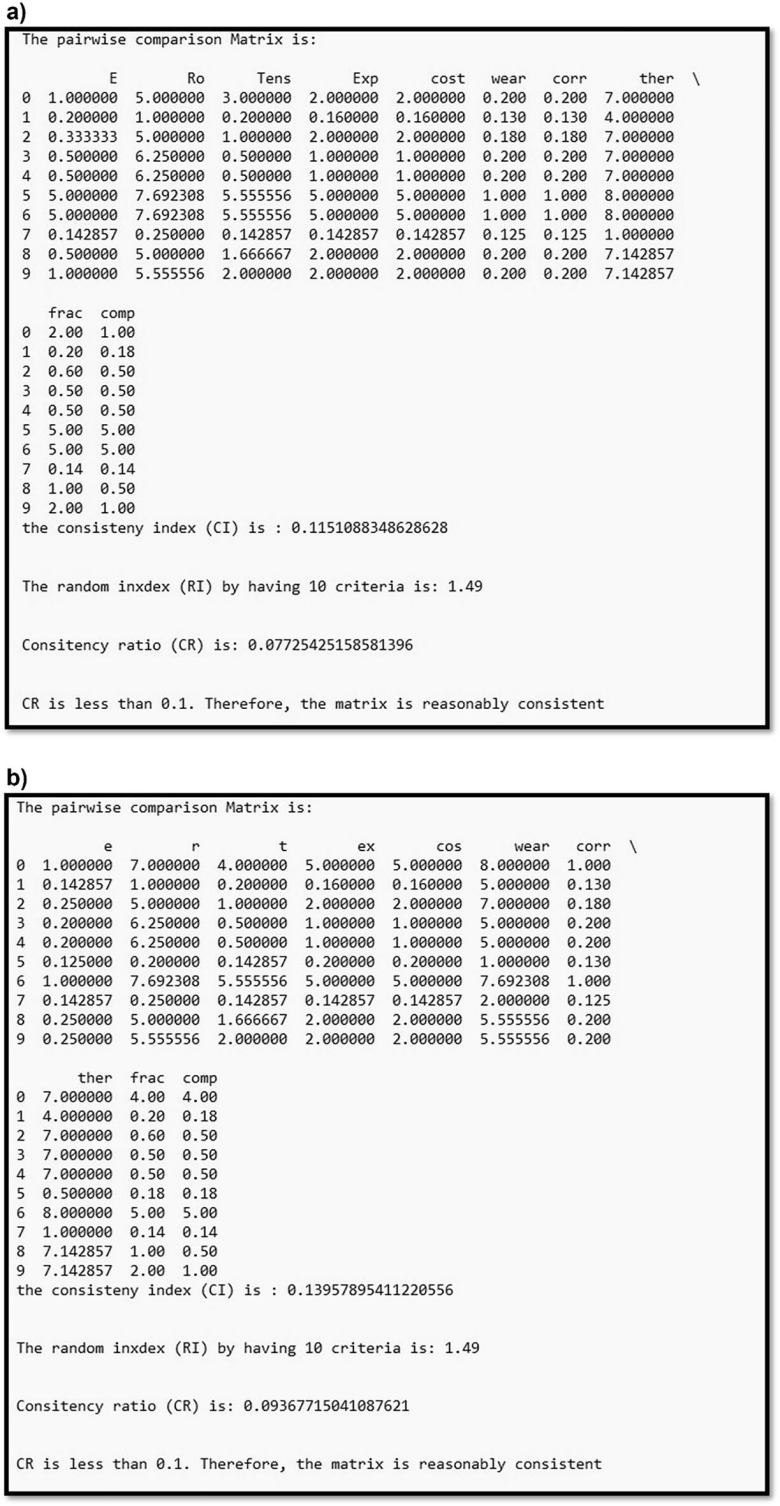
A part of the Python function produces a pair-wise comparison matrix, assesses consistency index, selects ratio index based on attribute count, and calculates consistency ratio for (**a**) articulating surfaces and (**b**) endplates. It compares the consistency ratio to the 0.1 threshold. If the values were below the threshold, a message says the pair-wise comparison matrix is reasonably consistent and evaluates fuzzy weight factors for each criterion. Otherwise, it requests an adjustment of the pair-wise comparison matrix until consistency is reached.

The calculations provided by the fuzzy AHP-TOPSIS methods were performed by developing a custom Python code (Python 3.8.5 programming language) in which all the data of materials and their properties were specified. The data can be introduced into the program manually or via a table file, depending on the preference. If the constructed pair-wise comparison matrix is inconsistent during the fuzzy AHP procedure, the program requests the adjustment of the pair-wise comparison matrix until consistency is achieved (Figs. 3, 4).

## Results

### Fuzzy AHP weighting factors

To establish normalized weights, the fuzzy AHP method was implemented. In this process, first an AHP pair-wise comparison matrix was determined as depicted in Table 3 for the design of the articulating surface and endplate.

According to the AHP pair-wise comparison matrices (Table 3), the consistency ratios for the articulating surface and endplate were 0.077 and 0.094, respectively (Fig. 4). This means that the consistency of each opinion was considered acceptable, with lower than the inconsistency threshold value (0.1).

The AHP pair-wise comparison matrices were transformed into TFN scales (according to Eq. 7) upon acceptance of the consistency ratio, and then the normalized fuzzy AHP weight factor was determined for each attribute (Table 6).

**Table 6:** The normalized fuzzy AHP weights for each criterion in design of (a) the articulating surface and (b) the endplate.

	Cw1	Cw2	Cw3	Cw4	Cw5	Cw6	Cw7	Cw8	Cw9	Cw10
(a)										
Normalized fuzzy AHP weights	0.095	0.018	0.063	0.058	0.058	0.264	0.264	0.012	0.076	0.092
(b)										
Normalized fuzzy AHP weights	0.245	0.025	0.086	0.070	0.070	0.015	0.269	0.015	0.095	0.110

In these tables, Cw1 = young’s modulus weight factor, Cw2 = density weight factor, Cw3 = tensile strength weight factor, Cw4 = expense of manufacturing process weight factor, Cw5 = cost of raw material weight factor, Cw6 = wear rate weight factor, Cw7 = corrosion resistance weight factor, Cw8 = thermal conductivity weight factor, Cw9 = fracture toughness weight factor, and Cw10 = compressive strength weight factor.

### Rank establishment by TOPSIS

All the criteria used for ranking the biomaterials had different units and dimensions. They need to be normalized by using Eq. (13). The normalized criteria values were then converted into normalized weighted values by multiplying with weights using Eq. (14). These normalized weight matrices for the design of the articulating surface and endplate are shown in Table 7.

**Table 7:** Normalized weighted matrix for design of (a) articulating surface and (b) endplate.

	E (GPa)	ρ (g/Cm^3^)	Tensile strength (MPa)	Expense of manufacturing process (qualitative)	Cost of raw material (qualitative)	Wear rate (qualitative)	Corrosion resistance (qualitative)	Thermal conductivity (w/mK)	Fracture toughness (MPa√m)	Compressive strength (MPa)
(a)										
ZTA	0.070	0.006	0.012	0.037	0.015	0.030	0.129	0.009	0.003	0.076
CoCr alloy	0.045	0.012	0.049	0.022	0.031	0.059	0.078	0.005	0.046	0.036
Ti–6Al–4V	0.023	0.006	0.033	0.022	0.023	0.089	0.104	0.003	0.042	0.032
Stainless steel 316L	0.039	0.011	0.017	0.015	0.008	0.118	0.052	0.006	0.044	0.017
UHMWPE	0.0002	0.001	0.0007	0.015	0.008	0.148	0.129	0.0002	0.003	0.0004
PEEK	0.0007	0.002	0.003	0.022	0.039	0.148	0.129	0.00009	0.003	0.003
(b)										
ZTA	0.180	0.008	0.017	0.045	0.019	0.002	0.132	0.011	0.003	0.091
CoCr alloy	0.117	0.016	0.067	0.027	0.037	0.003	0.079	0.007	0.057	0.043
Ti–6Al–4V	0.061	0.008	0.045	0.027	0.028	0.005	0.106	0.003	0.052	0.039
Stainless steel 316L	0.103	0.015	0.023	0.018	0.009	0.007	0.053	0.008	0.055	0.021
UHMWPE	0.0005	0.002	0.001	0.018	0.009	0.008	0.132	0.0002	0.004	0.0005
PEEK	0.002	0.002	0.004	0.027	0.047	0.008	0.132	0.0001	0.004	0.004

**Figure 5:**
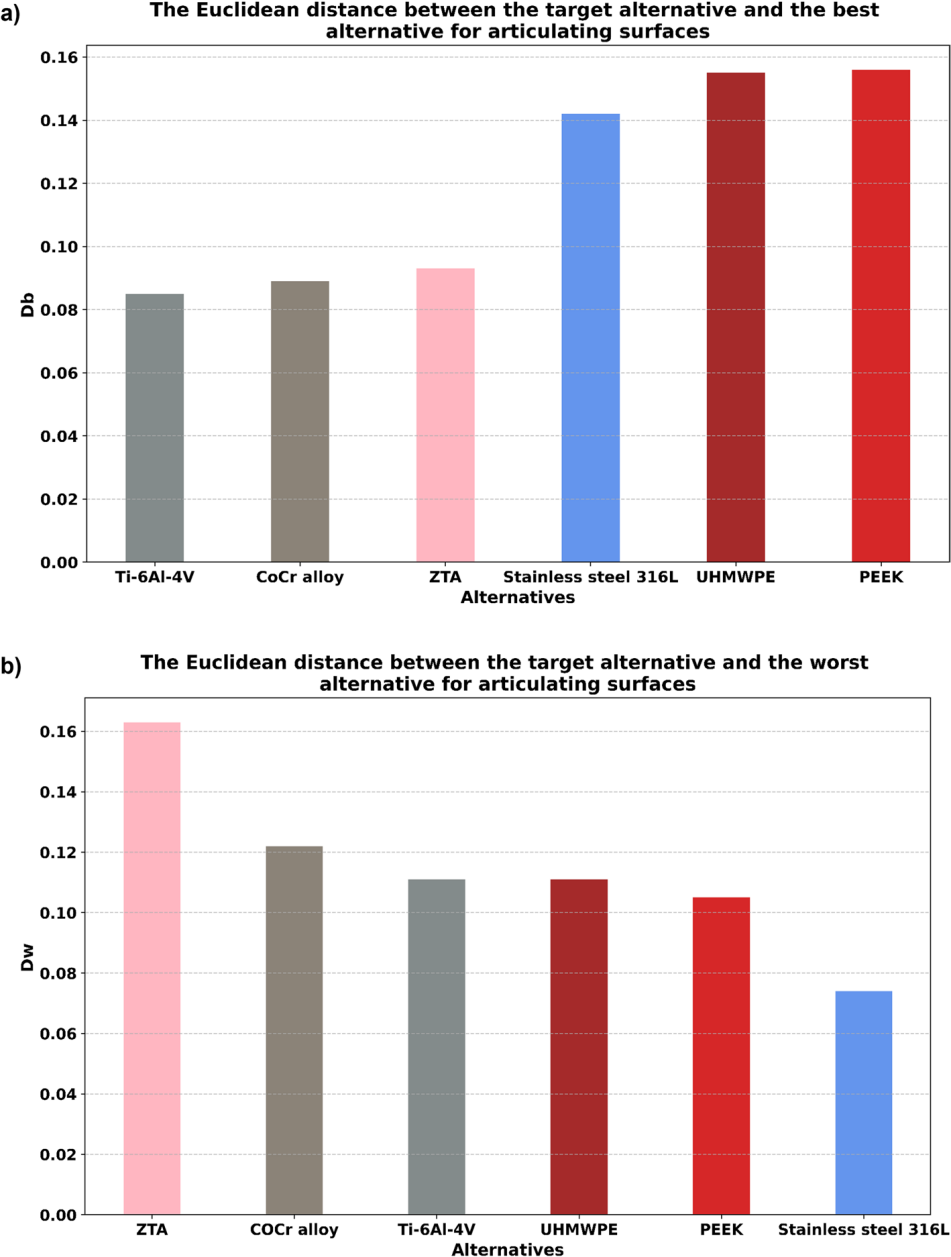
The Euclidean distance between the target alternative and (**a**) the best alternative, and (**b**) the worst alternative for articulating surfaces. The least distance to the best alternative and the greatest distance to the worst alternative are preferred, in that order.

**Figure 6:**
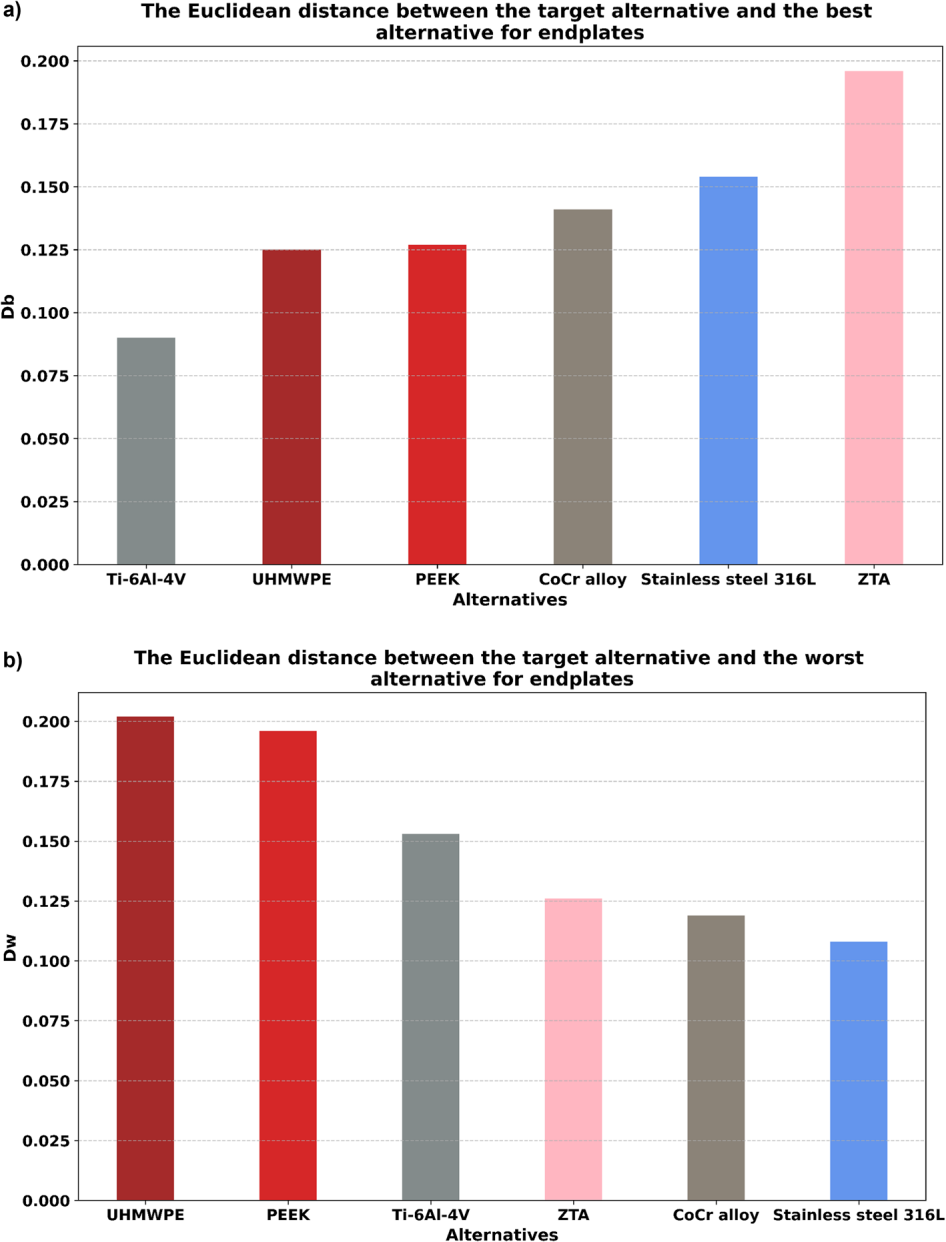
The Euclidean distance between the target alternative and (**a**) the best alternative and (**b**) the worst alternative for endplates. The least distance to the best alternative and the greatest distance to the worst alternative are preferred, in that order.

**Figure 7:**
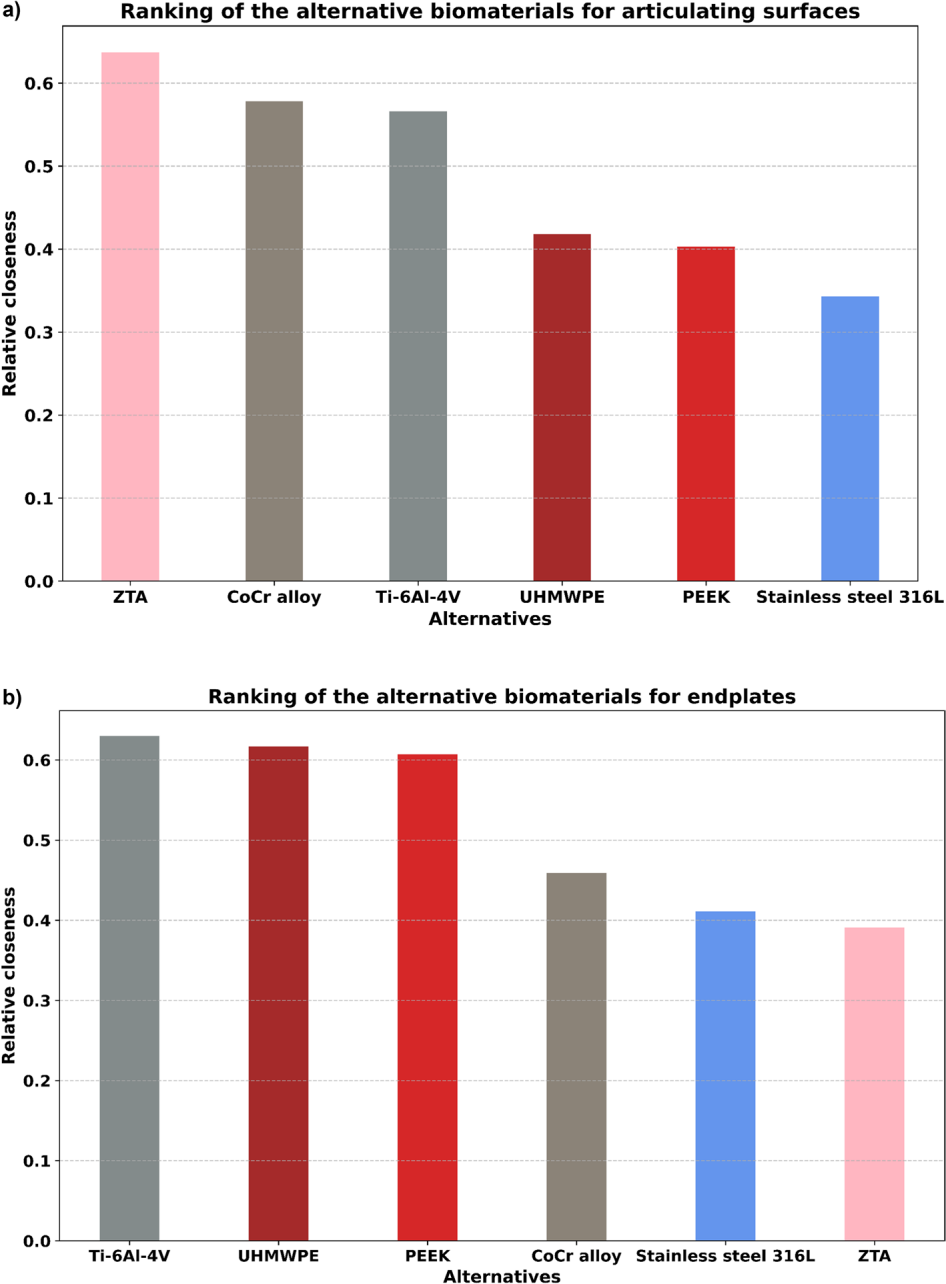
Ranking graph which is derived from the result of relative closeness for design of (**a**) articulating surface and (**b**) endplate in TDR applications.

After calculating the normalized weighted values, Eqs. (17)–(19) were used to calculate separation measures and relative closeness values (Table 8, Figs. 5, 6, 7). From these relative closeness values, the ranking was given, and the alternatives were prioritized (Table 8, Fig. 7). Based on the ranking results shown in Fig. 7 and Table 8, ZTA had the highest relative closeness value for the design of an articulating surface, whereas Ti–6Al–4V had the highest relative closeness value for the design of an endplate in TDR applications.

**Table 8:** The separation measures and the relative closeness for design of (a) articulating surface and (b) endplate.

	${D}_{i}^{b}$	${D}_{i}^{w}$	${S}_{i}$	Ranking
(a)				
ZTA	0.093	0.163	0.637	**1**
CoCr alloy	0.089	0.122	0.578	2
Ti–6Al–4V	0.085	0.111	0.566	3
Stainless steel 316L	0.142	0.074	0.343	6
UHMWPE	0.155	0.111	0.418	4
PEEK	0.156	0.105	0.403	5
(b)				
ZTA	0.196	0.126	0.391	6
CoCr alloy	0.141	0.119	0.459	4
Ti–6Al–4V	0.090	0.153	0.630	**1**
Stainless steel 316L	0.154	0.108	0.411	5
UHMWPE	0.125	0.202	0.617	2
PEEK	0.127	0.196	0.607	3

${{\text{D}}}_{{\text{i}}}^{{\text{b}}}, {{\text{D}}}_{{\text{i}}}^{{\text{w}}}$ and ${{\text{S}}}_{{\text{i}}}$ represent the best Euclidean distance, the worst Euclidean distance, and relative closeness respectively.

Bold emphasizes best ranked biomaterials for (a) articulating surface and (b) endplate.'

### Ranking validation and sensitivity analysis

The obtained ranking of proposed hybrid fuzzy AHP-TOPSIS was compared with fuzzy-VIKOR and fuzzy-MOORA approaches reported in Table 9. Accordingly, the ranking order for different alternative biomaterials was nearly similar when solved with other methods. Therefore, it can be concluded that proposed hybrid fuzzy AHP-TOPSIS method can be successfully implemented for selection of biomaterials based on given criteria with high accuracy. The slight observed variations can be attributed to data normalization techniques and mathematical foundations employed by each method.

**Table 9:** Comparison of fuzzy AHP-TOPSIS (proposed method) with fuzzy-VIKOR^56^ and fuzzy-MOORA^57^ methodologies.

	Fuzzy AHP-TOPSIS (proposed)	Fuzzy-VIKOR^56^	Fuzzy-MOORA^57^
(a)			
ZTA	**1**	**1**	**1**
CoCr alloy	2	3	3
Ti–6Al–4V	3	2	2
Stainless steel 316L	6	6	5
UHMWPE	4	4	4
PEEK	5	5	6
(b)			
ZTA	6	5	6
CoCr alloy	4	4	4
Ti–6Al–4V	**1**	2	**1**
Stainless steel 316L	5	6	5
UHMWPE	2	**1**	2
PEEK	3	3	3

Bold emphasizes best ranked biomaterials for (a) articulating surface and (b) endplate.

**Figure 8:**
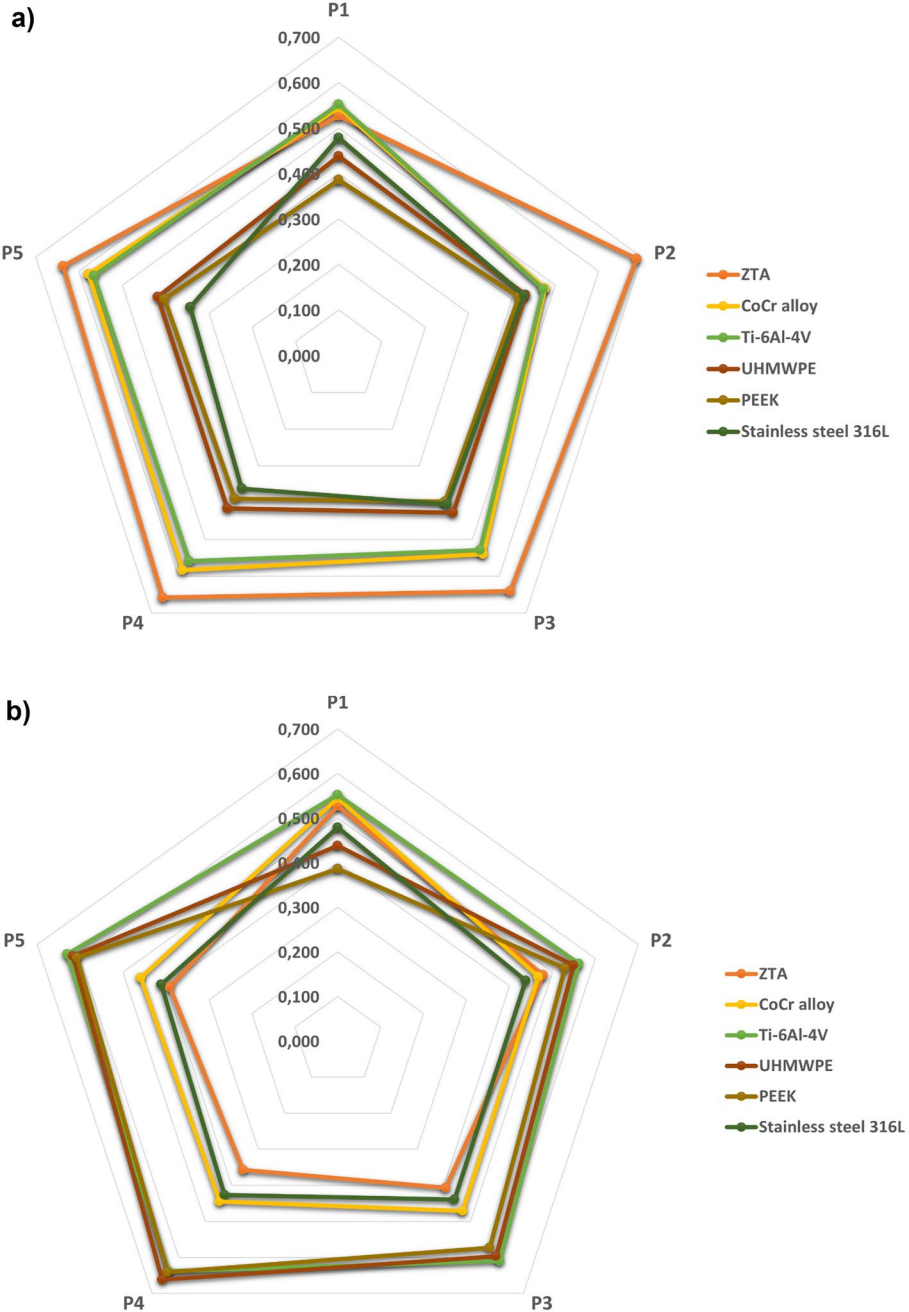
Sensitivity analysis for (**a**) articulating surface, and (**b**) endplate. p1 = equal weight factors, p2 = 33% most beneficial, 33% beneficial, and 33% least beneficial, p3 = 40% most beneficial, 40% beneficial, and 20% least beneficial, p4 = 50% most beneficial, 40% beneficial, and 10% least beneficial, and p5 = fuzzy AHP weight factors.

The sensitivity analysis was performed to further improve and validate the results of biomaterial selection provided by the proposed fuzzy AHP-TOPSIS method (Fig. 8, Table 10). From Fig. 8 and Table 10, it was evident that criteria weight variation can alter the ranking of each alternative. For an articulating surface (Fig. 8a, Table 10a), it was found that the A1 alternative (ZTA) had the highest relative closeness in 4 trials out of 5 experiments. In contrast, the A5 alternative (PEEK) and A6 alternative (stainless steel 316L) had the lowest relative closeness across most trials. For the endplate (Fig. 8a, Table 10a), it was found that the A3 alternative (Ti–6Al–4V) had the highest relative closeness in 4 trials out of 5 experiments. In contrast, the A1 alternative (ZTA) and A6 alternative (stainless steel 316L) had the lowest relative closeness across most trials. Hence, the sensitivity analysis justifies that the alternative A1 (ZTA) and alternative A3 (Ti–6Al–4V) can be selected as the top priority (80%) material for the design of the articulating surface and endplate, respectively. The final ranking is based on the outcomes of fuzzy AHP-TOPSIS and can be expressed as:

- A1 (ZTA) > A2 (CoCr alloy) > A3 (Ti–6Al–4V) > A4 (UHMWPE) > A5 (PEEK) > A6 (stainless steel 316 L) for articulating surface.
- A3 (Ti–6Al–4V) > A4 (UHMWPE) > A5 (PEEK) > A2 (CoCr alloy) > A6 (stainless steel 316 L) > A1 (ZTA) for endplate.

**Table 10:** Sensitivity analysis.

	Definition	Relative closeness						Ranking
(a)								
P1	Equal weight factors	A1	A2	A3	A4	A5	A6	A3 > A2 > A1 > A6 > A4 > A5
		0.527	0.545	**0**.**552**	0.438	0.386	0.479	
P2	33% most beneficial, 33% beneficial, and 33% least beneficial	A1	A2	A3	A4	A5	A6	A1 > A2 > A3 > A4 > A6 > A5
		**0**.**688**	0.477	0.473	0.432	0.416	0.428	
P3	40% most beneficial, 40% beneficial, and 20% least beneficial	A1	A2	A3	A4	A5	A6	A1 > A2 > A3 > A4 > A6 > A5
		**0**.**641**	0.540	0.529	0.427	0.397	0.405	
P4	50% most beneficial, 40% beneficial, and 10% least beneficial	A1	A2	A3	A4	A5	A6	A1 > A2 > A3 > A4 > A5 > A6
		**0**.**658**	0.584	0.559	0.416	0.389	0.362	
P5	Fuzzy AHP weight factors	A1	A2	A3	A4	A5	A6	A1 > A2 > A3 > A4 > A5 > A6
		**0**.**637**	0.578	0.566	0.418	0.403	0.343	
(b)								
P1	Equal weight factors	A1	A2	A3	A4	A5	A6	A3 > A2 > A1 > A6 > A4 > A5
		0.527	0.545	**0**.**552**	0.438	0.386	0.479	
P2	33% most beneficial, 33% beneficial, and 33% least beneficial	A1	A2	A3	A4	A5	A6	A3 > A4 > A5 > A1 > A2 > A6
		0.479	0.467	**0**.**562**	0.549	0.529	0.438	
P3	40% most beneficial, 40% beneficial, and 20% least beneficial	A1	A2	A3	A4	A5	A6	A3 > A4 > A5 > A2 > A6 > A1
		0.407	0.471	**0**.**610**	0.597	0.573	0.440	
P4	50% most beneficial, 40% beneficial, and 10% least beneficial	A1	A2	A3	A4	A5	A6	A4 > A5 > A3 > A2 > A6 > A1
		0.357	0.445	0.638	**0.662**	0.641	0.427	
P5	Fuzzy AHP weight factors	A1	A2	A3	A4	A5	A6	A3 > A4 > A5 > A2 > A6 > A1
		0.391	0.459	**0.630**	0.617	0.607	0.411	

A1 = ZTA, A2 = CoCr alloy, A3 = Ti–6Al–4V, A4 = UHMWPE, A5 = PEEK, A6 = stainless steel 316L.

Bold emphasizes maximum relative closeness value for different criteria weights.

According to the ranking validation and sensitivity analysis, it can be confirmed that hybrid fuzzy AHP-TOPSIS is effective and robust in material selection for spinal disc implants.

## Discussion

TDR typically achieves motion preservation by articulating surfaces, which are susceptible to wear under repetitive motion and loading^58^. Polymeric debris generated by metal on polymer bearing surfaces can result in osteolysis and implant loosening^59^. While metal on metal bearing surfaces have superior wear properties, high metal concentrations and nanodebris can lead to local and systemic effects^59^. Ceramic on ceramic bearing surfaces showed less wear debris with lower biological reactivity due to their outstanding tribological properties and excellent biocompatibility^60^. In this study, ZTA also proved to be the optimal material for designing articulating surfaces in TDR applications. The most prevalent metallic biomaterials, namely CoCr alloy and Ti–6Al–4V, were afterwards ranked second and third, respectively. Even though ZTA has better biocompatibility and tribological qualities than CoCr alloy and Ti–6Al–4V, these materials were relatively close (Fig. 7, Table 8). It was justifiable that the technique offered a tradeoff between desired parameters. In this instance, ZTA’s lower fracture toughness, greater Young’s modulus, and more expensive manufacturing process cost contributed to a similar performance score for the design of articulating surfaces as compared to CoCr alloy and Ti–6Al–4V.

The higher Young’s modulus of biomaterials compared to cortical bone can lead to stress shielding, the remodeling-induced reduction of bone due to the removal of mechanical stress from the bone by an implant^61^. It is essential to achieve initial and long-term stability at the bone-implant interface to avoid these complications by considering materials with similar modulus to bone, adequate osseointegration and osseoconductivity, high fracture toughness, and corrosion resistance. In this investigation, Ti–6Al–4V was demonstrated to be the optimal alternative material for designing TDR endplates. Then, UHMWPE and PEEK followed closely behind Ti–6Al–4V in terms of performance. Although PEEK’s elastic modulus was comparable to that of cortical bone, it was ranked third in this study. This placement was influenced by the PEEK’s greater cost of raw materials, lower fracture toughness, compressive and tensile strengths. However, the difference in relative closeness between PEEK and Ti–6Al–4V was not considerable, and they can be utilized interchangeably (Fig. 7, Table 8). It was somewhat unexpected that ZTA came in last in the ranking. The ZTA ranking could be justified by the material’s high Young’s modulus, high cost, and low fracture toughness.

In addition to the factors outlined in Table 1, osseointegration, CT and MRI compatibility, and the risk of implant-related infections play key roles in the selection of biomaterials. Lee et al.^62^ conducted an in-vitro evaluation of the biological response of cells to different biomaterials, including ZTA, PEEK, silicon nitride (SN) and surface-textured silicon nitride (ST-SN) and Ti–6Al–4V. compared to Ti–6Al–4V, they found that all other materials generally demonstrated lower osteoclastic activity and inflammatory response. They also demonstrated that ZTA and SN enhanced osteogenic differentiation and actin length^62^. Moreover, ceramic biomaterials are more compatible with CT and MRI than metals, which can result in problematic artefacts, or frequently used polymers that are radiolucent. Consideration of these characteristics in the decision-making process will alter the relative closeness of biomaterials, particularly for ZTA. Notably, only a small number of these characteristics are available for several of the materials evaluated in this work. Therefore, it is recommended to undertake more in-vitro research addressing the evaluation of material biocompatibility and include the results in the process of material selection.

The decision-making procedure revealed that the biomaterial ranking varies depending on the function. According to this approach, the combination of these materials could be effective in reducing clinical issues including inflammatory response, heterotopic ossification, dislocation, and migration. However, this combination must be technologically feasible for production. Recently, the Simplify® Cervical Artificial Disc has completed review for premarket approval for use in patients suffering from radiculopathy or myelopathy^63^. This implant was composed of two PEEK endplates and a ceramic core (ZTA). The external surface of the endplates was coated with titanium to promote bone formation and facilitate attachment to the vertebrae located above and below the implant. The materials employed in this implant were consistent with the outcomes of fuzzy AHP-TOPSIS. However, longitudinal clinical evidence is required for a complete knowledge of the efficacy of both the logical method and the implant.

Ghaleb et al.^67^ evaluated numerous alternatives based on process agility, computing complexity, and the number of possible processes and criteria for manufacturing process application. Based on computational complexity, they determined that VIKOR outperformed TOPSIS and AHP. In terms of decision-making agility, the TOPSIS and VIKOR approaches were more applicable, although the rankings derived by AHP, TOPSIS, and VIKOR for the selection of manufacturing processes were nearly identical^67^. In our study, the proposed fuzzy AHP-TOPSIS outcome was also compared with fuzzy-VIKOR and fuzzy-MOORA methods (Table 9). Accordingly, ZTA obtained the highest ranking across all the approaches for the selection of the articulating surface material. In terms of the endplate, Ti–6Al–4V exhibited the highest ranking according to the fuzzy AHP-TOPSIS and fuzzy MOORA methodologies. However, it obtained the second highest ranking according to fuzzy-VIKOR method, indicating a nearly same ranking across all approaches. Furthermore, the sensitivity analysis was performed to validate the outcomes obtained by the fuzzy AHP-TOPSIS method. When the results of the sensitivity analysis were evaluated collectively (Fig. 8, Table 10), they were overall consistent with one another. Nonetheless, a moderate difference was observed, revealing that the opinions of experts could affect the outcomes.

In the literature, several different approaches have been proposed for material selection, including MOORA, AHP, COPRAS, VIKOR, and TOPSIS. However, preference ranking organization method for enrichment evaluation (PROMETHEE) is one of a new ranking method which is considered as simple in conception and computation compared to many other MCDM methods^64^. PROMETHEE focuses on pair-wise comparisons to establish outranking relationships between alternatives and facilitates both partial and complete ranking of alternatives^65^. It also provides a superior visual representation during the concluding phase of the problem-solving process in comparison to alternative MCDM methods like AHP^66^. Several MCDM methods necessitate significantly more inputs than PROMETHEE^66^. For future research, it is advisable to employ the PROMETHEE approach to prioritize spinal implant biomaterials and subsequently compare the obtained findings with those derived from other MCDM methods, such as fuzzy AHP-TOPSIS.

## Conclusion

This study emphasized the necessity of a unique and justified selection of biomaterials in accordance with their intended purpose during the early stage of development. The technique enables the consideration of multiple qualitative and quantitative criteria throughout the material selection process for a particular design. Moreover, it avoids later costs and delays and generates ideas through a systematic search of biomaterials. However, the assignment of acceptable numerical values for qualitative criteria necessitates a review of the relevant literature and consultation with industry sector experts in order to minimize bias or individual opinion in the results. Furthermore, it requires a thoughtful selection of criteria, as this selection also has a direct impact on the outcome.

## Data Availability

All data generated and analyzed in this study are included in this published article.
